# Development and performance evaluation of the Medicines Optimisation Assessment Tool (MOAT): a prognostic model to target hospital pharmacists’ input to prevent medication-related problems

**DOI:** 10.1136/bmjqs-2018-008335

**Published:** 2019-03-07

**Authors:** Cathy Geeson, Li Wei, Bryony Dean Franklin

**Affiliations:** 1 Pharmacy, Luton and Dunstable University Hospital NHS Foundation Trust, Luton, UK; 2 Research Department of Practice and Policy, UCL School of Pharmacy, London, UK; 3 Pharmacy Department, Imperial College Healthcare NHS Trust, Charing Cross Hospital, London, UK

**Keywords:** medication safety, adverse events, epidemiology and detection, decision support, clinical, health services research, pharmacists

## Abstract

**Background:**

Medicines optimisation is a key role for hospital pharmacists, but with ever-increasing demands on services, there is a need to increase efficiency while maintaining patient safety.

**Objective:**

To develop a prediction tool, the Medicines Optimisation Assessment Tool (MOAT), to target patients most in need of pharmacists’ input in hospital.

**Methods:**

Patients from adult medical wards at two UK hospitals were prospectively included into this cohort study. Data on medication-related problems (MRPs) were collected by pharmacists at the study sites as part of their routine daily clinical assessments. Data on potential risk factors, such as number of comorbidities and use of ‘high-risk’ medicines, were collected retrospectively. Multivariable logistic regression modelling was used to determine the relationship between risk factors and the study outcome: preventable MRPs that were at least moderate in severity. The model was internally validated and a simplified electronic scoring system developed.

**Results:**

Among 1503 eligible admissions, 610 (40.6%) experienced the study outcome. Eighteen risk factors were preselected for MOAT development, with 11 variables retained in the final model. The MOAT demonstrated fair predictive performance (concordance index 0.66) and good calibration. Two clinically relevant decision thresholds (ie, the minimum predicted risk probabilities to justify pharmacists’ input) were selected, with sensitivities of 90% and 66% (specificity 30% and 61%); these equate to positive predictive values of 47% and 54%, respectively. Decision curve analysis suggests that the MOAT has potential value in clinical practice in guiding decision-making.

**Conclusion:**

The MOAT has potential to predict those patients most at risk of moderate or severe preventable MRPs, experienced by 41% of admissions. External validation is now required to establish predictive accuracy in a new group of patients.

## Introduction

Medicines are the most common intervention in healthcare.^1^ However, there is growing evidence of a need to improve medicines use.^1–7^ Medication safety is high on international and national agendas, with recent publication of the WHO’s third Global Patient Safety Challenge: Medication Without Harm,^7^ and a recent report on the prevalence and economic burden of medication errors in the English National Health Service.^8^ While the majority of medicine use occurs in primary care, safe use of medicines in secondary care and at transitions of care continue to be areas of concern,^7 8^ together with ongoing calls for hospital pharmacy services to operate more efficiently.^9–14^

Clinical prioritisation has been proposed as a way to permit pharmacy services to focus on those in greatest need and where clinical pharmacy input is likely to have greatest impact, requiring a method to triage patients to assign ‘pharmaceutical acuity’.^14 15^ Prediction tools to identify hospitalised patients at risk of adverse medication-related outcomes have previously been developed,^16–27^ but the majority identify patients at risk of adverse drug reactions,^17–19^ adverse drug events^20 21^ or medication errors^22^ in isolation and/or are based on ‘expert opinion’ rather than statistical determination.^23–27^

This study therefore aimed to develop a methodologically sound prognostic model,^28–31^ the Medicines Optimisation Assessment Tool (MOAT), to identify hospital patients most in need of pharmacists’ input based on their risk of moderate or severe preventable medication-related problems (MRPs). Our objectives were to develop a decision aid for use in clinical practice to allocate patients to risk groups and to assess its predictive performance and clinical usefulness.

## Method and analysis

### Study design

The MOAT was developed using a prospective cohort study involving adults admitted to the medical wards of two hospitals in South East England, described in detail in a published protocol.^32^ Eligible patients were consecutively included at Hospital A from 28 April 2016 to 31 May 2016 and Hospital B from 19 October 2016 to 1 November 2016. As previously described,^32^ patients admitted for investigation only (ie, elective admissions), and those not prescribed medication during the admission, were excluded on the basis that they did not represent the target population for the MOAT. Patients were also excluded if their prescribing records were not reviewed by a clinical pharmacist during their admission, as it was not possible to ascertain whether they experienced an MRP. In addition to these previously published exclusion criteria, patients were also excluded if their prescribing records and/or medical notes were unavailable; this was to ensure completeness of medicine-related predictor data. MRP data were collected for all study patients from admission to discharge from hospital or the date the study closed (2 weeks after inclusion of the final patient), whichever occurred sooner.

Descriptions of the outcome for the prognostic modelling, preselected candidate predictors and methods of data collection are described in detail elsewhere.^32^ In summary, the outcome event was the occurrence of at least one moderate or severe preventable MRP, chosen on the basis that prioritisation would be required for patients at risk of moderate or severe MRPs irrespective of the number. MRPs were defined as ‘all circumstances involving a patient’s drug treatment that actually, or potentially, interfere with the achievement of an optimal outcome’.^33^ Severity was assessed by an expert panel comprising the principal investigator, a hospital pharmacist, a senior nurse and a consultant physician using a validated visual analogue scale.^34^ Preventability was assessed at the point of identification and expressed as a dichotomous variable of yes or no. Further information on study outcomes, including illustrative examples, is given in online supplementary appendix S1. We focused on *moderate or severe* MRPs as these are most clinically relevant. Similarly, we focused on *preventable* MRPs to ensure the MOAT identifies patients with MRPs amenable to pharmacist intervention. Eighteen candidate predictors were preselected (online supplementary appendix S1): age, socioeconomic status, previous allergy, body mass index, number of previous hospital admissions, primary diagnosis, number of comorbidities, history of dementia, number of medicines prescribed, use of one or more of a list of ‘high-risk medicines’, parenteral medicine administration, renal function, liver disease, serum albumin, serum potassium, serum sodium, white cell count and platelet count. The following changes were made to the proposed candidate predictors following publication of the protocol:^32^

10.1136/bmjqs-2018-008335.supp1 Supplementary data Supplementary information on methods

- an organ-based approach^35^ was used to categorise primary diagnosis rather than the proposed International Classification of Diseases system. This was to reduce the risk of misclassification given that a definitive diagnosis may not be known at the point of hospital admission. This resulted in eight categories: cardiovascular, respiratory, gastrointestinal, genitourinary, musculoskeletal-integumentary, endocrine-metabolic, nervous system/mental disorders and ‘other’ (all other diagnoses combined);
- the high-risk medicine category ‘antibiotics’ was changed to ‘antimicrobial agents’ to include antivirals, antifungals and antiprotozoal agents as well as antibiotics;
- renal function was estimated using the modified Modification of Diet in Renal Disease equation rather than the Cockcroft-Gault equation.^36^ This was due to limited availability of patient weight data.

Data entry checks for the accuracy of candidate predictor data were performed on a randomly selected 10% sample of patients. Sixteen data items were checked for each of these patients, and accuracy calculated as the percentage of data items recorded correctly. We also developed and used a ‘MRP identification assessment exercise’ to quantify potential variability in MRP identification between pharmacists at the study sites. This involved the use of four fictitious medication charts each including three or four MRPs. Each MRP was considered to have a binary outcome in terms of whether or not it was identified by each pharmacist. The percentage agreement between pharmacists was calculated and Randolph’s kappa used to assess chance-adjusted agreement (online supplementary appendix S1).

The sample size was dictated by practical considerations, permitting inclusion of 1500 patients plus 10% to allow for patient exclusions.^32^ Adequacy was assessed using the ‘events per variable’ (EPV)^37^ and precision methods, based on a conservative estimate for the outcome prevalence of 32%, obtained following pilot work with 200 patients. This gave an anticipated EPV of 13, exceeding the ‘rule of thumb’ of 10 or more EPV,^37^ and the ability to estimate the MOAT’s sensitivity with 95% CI of ±3%, which we considered an acceptable level of precision in terms of clinical usefulness.

### Data analysis

Data analysis was performed as specified in the protocol to reduce the risk of data-driven model development.^32^ All continuous predictors were analysed as such; we did not use categorisation as this is associated with reduced model reliability and overoptimistic predictive performance.^31^ Predictors with a wide range in units were analysed as deciles to aid comparison of predictive effects among variables (age, estimated glomerular filtration rate, platelet count and the percentage deprivation rank used as a measure of socioeconomic status); changes in predictive effect per unit increase would otherwise be small, making comparison with other predictors more difficult.^38^ Missing candidate predictor data were handled using multiple imputation and truncation used to reduce the influence of outliers on the regression coefficients;^38^ truncation was chosen instead of data transformation due to the potential impact of transformations on interpretability of the MOAT.^39^ Further information on missing data and truncation are given in Online supplementary appendix S1. Linearity of continuous predictors was checked using multivariable fractional polynomial modelling;^40 41^ this failed to reject linear relationships for the continuous predictors, and data transformations were therefore not required. There was also no evidence of multicollinearity (assessed by calculating variance inflation factors).^38^ Exploratory investigations into possible interactions between predictors were not performed as none were hypothesised a priori, while interactions may have been present, thorough assessment of possible interactions during modelling increases the risk of overfitting and does not necessarily increase prognostic model performance.^38 42 43^

Model development is described in online supplementary appendix S1. In summary, a random effects model was used to account for possible correlation between patients admitted more than once during the study period.^44^ Backwards elimination was used to reduce the set of candidate predictors during modelling as our aim was to produce a parsimonious model, thereby increasing clinical applicability while retaining reasonable predictive performance.^38^

Internal validation involved the use of 200 bootstrap samples (online supplementary appendix S1).^38 42 45^ This involved drawing random samples from the developmental dataset and constructing a model, similar to the original regression model, in each random sample. Each bootstrap model was then applied to the original developmental dataset, and optimism calculated as the average difference in performance, in terms of the concordance index (c-index) and calibration slope, between the bootstrap and developmental datasets. Bootstrap validation suggested slight overfitting, the model was therefore adjusted for optimism by multiplying each of the model’s regression coefficients by a ‘linear shrinkage factor’; the adjusted model was then used to create an electronic scoring system. Our original proposal was to develop a simplified scoring system by converting the regression coefficients from the final prognostic model into scores,^46^ but an electronic system permitted use of the full regression equation, so preventing loss of predictive accuracy, simplifying use and reducing the risk of calculation errors. It also permitted incorporation of usage instructions.

While prognostic models provide estimates of the probability that an individual patient will experience an outcome event, this does not provide guidance on an appropriate course of action. ‘Risk groups’ are therefore often created, which indicate a specific course of action, creating a ‘decision aid’ or ‘clinical decision rule’.^38^ We created three risk groups, categorising patients as low, medium or high risk. The choice of decision thresholds, which are the cut-offs for predicted risk probabilities to justify an intervention (in this case, pharmacists’ input), was guided by a survey of healthcare professionals and patient/public representatives, and consensus views of practising pharmacy staff. This is described further in online supplementary appendix S1. As concern exists over the arbitrary nature of categorisation, with all patients within a group being assumed to have the same risk,^42^ we chose to report both the risk group and individual predicted risk probability for each patient assessed using the MOAT. This was to guide general prioritisation decisions (by categorising patients as high, medium or low-risk) and also to permit some degree of prioritisation within each category if required.

### Assessment of clinical usefulness and credibility

Clinical usefulness was assessed using decision curve analysis,^38 47^ which assesses model performance over a range of decision thresholds using the theoretical relationship between threshold probabilities and the relative value of false positive and false negative results,^48^ calculated as the net benefit (online supplementary appendix S1). By varying the threshold probability, it is possible to produce a ‘decision curve’ (a plot of net benefit against threshold probability), which informs the range of threshold probabilities for which the prediction model would be of value in clinical practice.^49^

Adoption of a prediction tool into clinical practice requires clinical credibility,^50^ which is based on factors such as content validity, ease of use, acceptability of the time taken to use the tool and acceptability of the false negative rate.^51 52^ To investigate these we used: (1) a consensus method to harness the insights of pharmacy professionals regarding the perceived clinical credibility and usability of the MOAT; (2) an assessment of the workload implications and (3) an assessment of the clinical implications of false negative predictions (described in online supplementary appendix S1).

Results are reported according to the Transparent Reporting of a multivariable prediction model for Individual Prognosis Or Diagnosis (TRIPOD) reporting guidelines for prognostic model studies,^30^ and Strengthening the Reporting of Observational Studies in Epidemiology reporting guidelines for observational studies.^53^ All analyses were conducted using Stata V.14.2.

## Results

### Overview of included patients

**Figure 1:**
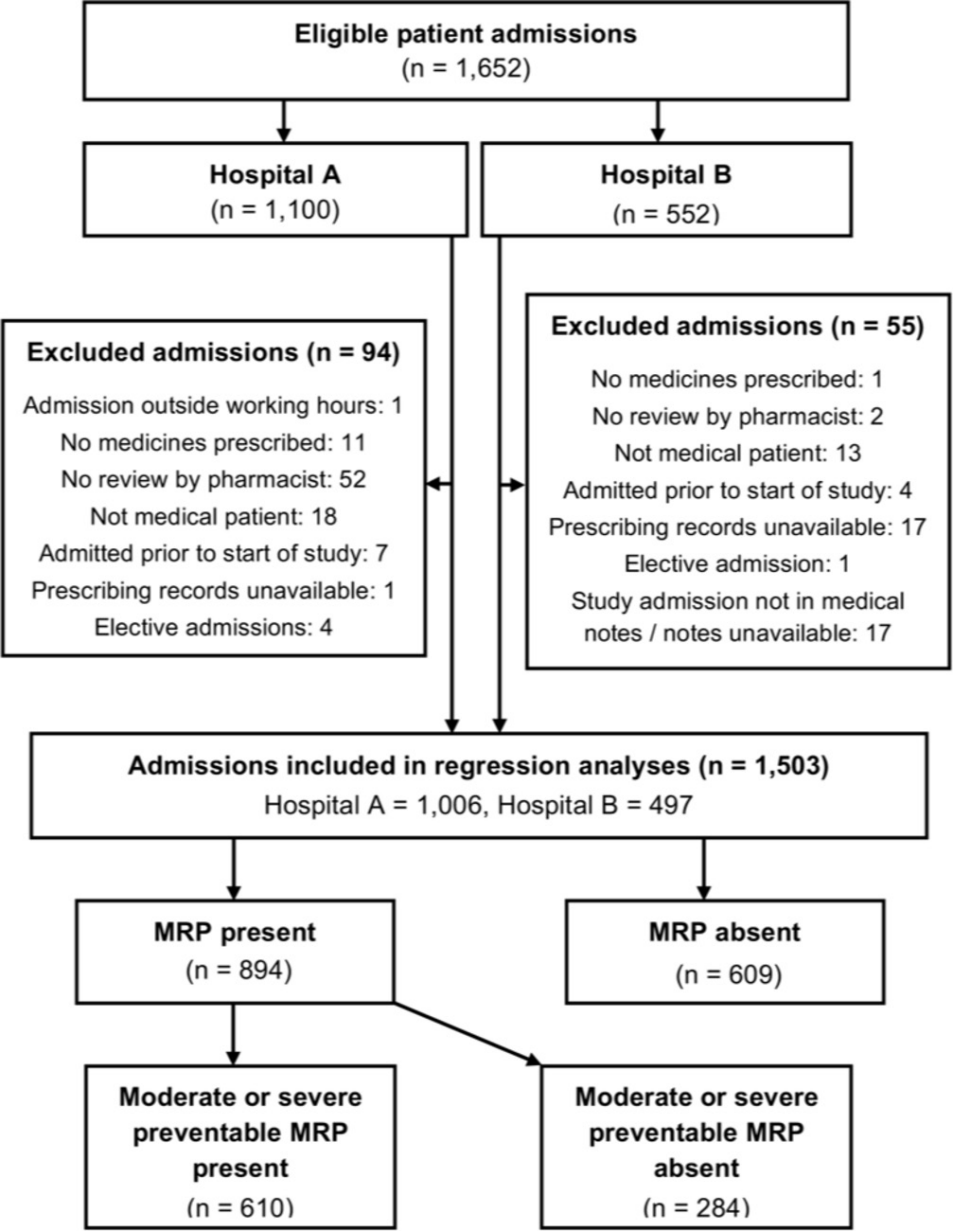
Participant flow diagram. MRP, medication-related problems.

In total, 1652 patient admissions were included in the study: 1100 from Hospital A and 552 from Hospital B (figure 1). Of these admissions, 149 (9%) were excluded: 114 did not meet the eligibility criteria, and prescribing records and/or medical records were unavailable for 35. Online supplementary appendix S2 provides further information of this and other online supplementary results. Of the remaining 1503 admissions, 1378 (92%) were followed up until discharge from hospital: 933 (93%) of 1006 at Hospital A and 445 (90%) of 497 at Hospital B. The remaining 125 admissions were followed until the study end date (2 weeks after inclusion of the final patient at each site). Fifty-seven patients entered the study twice: 46 at Hospital A and 11 at Hospital B. One further patient at Hospital B entered the study three times. The total number of unique patients included in the study was therefore 1444 (960 at Hospital A and 484 at Hospital B). Of the 1503 admissions, 894 (59.5%) experienced at least one MRP, with 610 (40.6%) experiencing the outcome event, namely at least one moderate or severe preventable MRP.

10.1136/bmjqs-2018-008335.supp2 Supplementary data Supplementary information on results

Key characteristics of the 1503 study admissions are summarised in table 1.

**Table 1:** Characteristics of study admissions

Characteristic	Admissions=1503 Mean/median / n (% of admissions)	Missing values n (% of admissions)
Demographic		
Age (years)	Median: 75 IQR: 58–85	0
Gender (female)	693 (46.1%)	0
Socioeconomic status, ranked using English Indices of Deprivation 2015 – Index of Multiple Deprivation^75^ *	Median: 50† IQR: 30–79	6 (0.4%)
Ethnic origin (white)	1208 (85.9%)†	96 (6.4%)
Patient related		
Previous allergy	582 (38.8%)†	1 (0.07%)
Body mass index (kg/m^2^; healthy weight range 18.5–24.9)	Median: 24.9† IQR: 21.4–29.1	341 (22.7%)
Number of hospital admissions in previous 6 months	Median: 0 IQR: 0–1	0
Primary diagnosis:		
Endocrine and metabolic	82 (5.5%)	0
Nervous system and mental disorders	149 (9.9%)	0
Cardiovascular system	315 (21.0%)	0
Respiratory system	332 (22.1%)	0
Gastrointestinal system	144 (9.6%)	0
Genitourinary system	144 (9.6%)	0
Musculoskeletal-integumentary systems	93 (6.2%)	0
All other categories	244 (16.2%)	0
Number of comorbidities	Median: 4 IQR: 2–5	0
History of dementia	161 (10.7%)	0
Length of hospital stay (days)	Median: 5 IQR: 2–12	0
Medicines related		
Medicines reconciliation completed	1292 (86.0%)	0
Number of medicines‡	Median: 8 IQR: 5–10	0
Parenteral medicines administration	1008 (67.1%)	0
Use of high-risk medicines:		
Systemic antimicrobials (excluding aminoglycosides and glycopeptides)	937 (62.3%)	0
Antidepressants	351 (23.4%)	0
Anticoagulants	312 (20.8%)	0
Antidiabetic medication	299 (19.9%)	0
Epilepsy medicines	227 (15.1%)	0
Therapeutic heparin	222 (14.8%)	0
Antiarrhythmics	150 (10.0%)	0
Opioids	145 (9.6%)	0
Aminoglycosides and glycopeptides	105 (7.0%)	0
Antipsychotics (excluding clozapine)	92 (6.1%)	0
Other high-risk medicines (clozapine, antiretrovirals, medicines for Parkinson’s disease)	40 (2.7%)	0
Theophylline and aminophylline	38 (2.5%)	0
Immunosuppressants	21 (1.4%)	0
Cytotoxics	14 (0.9%)	0
Lithium	6 (0.4%)	0
Laboratory results		
Renal function—estimated glomerular filtration rate (mL/min/1.73 m^2^; normal >90)§	Median: 73† IQR: 53–99	9 (0.6%)
Liver disease¶	164 (10.9%)	0
Serum albumin (g/L; reference range 35–50)	Mean: 33.0† SD: 6.0	26 (1.7%)
Serum potassium (mmol/L; reference range 3.5–5.3)	Mean: 4.4† SD: 0.62	30 (2.0%)
Serum sodium (mmol/L; reference range 133–156)	Mean: 137.2† SD: 5.2	3 (0.2%)
White cell count (10^9^/L; reference range 3.2–11.0)	Median: 9.8† IQR: 7.5–12.8	6 (0.4%)
Platelet count (10^9^/L; reference range 120–450)	Median: 244† IQR: 192–312	8 (0.5%)

*Deprivation rank based on patients’ postcode, shown as the ranked position as a percentage of all neighbourhoods in England (where one is the most deprived).

†Results for patients without missing data.

‡Number of ‘regular’ medicines prescribed on the first full day of admission to hospital (ie, excluding ‘when required’ and ‘once only’ medicines, dietary products, non-medicated topical products, wound dressings).

§Glomerular filtration rate estimated using modified Modification of Diet in Renal Disease equation.^36^

¶Liver disease defined as alanine aminotransferase/alkaline phosphatase and/or bilirubin ≥3 times normal range and/or documented liver disease.

Data entry checks were performed for 152 admissions (99 from Hospital A and 53 from Hospital B), giving a total of 2432 data item checks; 2429 (99.9%) were correctly recorded, with remedial action taken to refine data entry where necessary.

### MRP identification assessment exercise

The overall percentage agreement (proportion of MRPs identified) by 59 pharmacists from the study sites was 84.5%, with a Randolph’s kappa coefficient of 0.50 suggesting ‘moderate agreement’.^54 55^ Further details are given in online supplementary appendix S2.

### Exploratory data analysis

A review of the distributions of the preselected categorical predictors identified five categories each representing fewer than 5% of the study population: theophylline and aminophylline, immunosuppressants, cytotoxics, lithium and ‘other high-risk medicines’ (table 1). As all were categories of high-risk medicines, these categories were combined to create a larger ‘other high-risk medicines’ category. Clozapine was also moved from ‘other high-risk medicines’ to ‘antipsychotics’ on the basis that it is more closely related in terms of pharmacological use.

Truncation was required to reduce the influence of outliers for seven variables, and analysis of missing predictor data supported use of multiple imputation (see online supplementary appendix S2).

### Adequacy of sample size

Following data collection, it was possible to review the adequacy of the sample size. Two changes affected the EPV calculation: the increased outcome prevalence compared with the initial estimate and the reduced number of variables. As non-linear transformations were not required and no interactions between predictors examined, this resulted in an increase in the EPV to 18. The higher number of outcome events also led to increased precision in estimation of the MOAT’s sensitivity compared with our initial estimate.^32^

### Model building

Thirteen predictors were retained in the model following backward elimination: socioeconomic status, number of comorbidities, number of medicines, estimated glomerular filtration rate, white cell count, previous allergy, systemic aminoglycosides and glycopeptides, other systemic antimicrobials, epilepsy medicines, antidepressants and three primary diagnoses (nervous system/mental disorders, respiratory and gastrointestinal). After considering the sensibility of using these predictors, we excluded socioeconomic status due to: (1) its relative complexity, with potential to reduce ease of use of the MOAT in clinical practice; (2) recognition that inclusion may reduce the generalisability of the MOAT by restricting use to English hospitals; (3) the minimal impact of removal on the model’s c-index (0.3% reduction). Once socioeconomic status was excluded from the model, ‘antidepressants’ became non-significant and was therefore also excluded, leaving 11 predictors in the final model (table 2).

**Table 2:** Multivariable association between predictors and outcome events after correction for optimism, including the model constant

Predictor	Adjusted regression coefficient*† (95% CI)	P value‡
Number of comorbidities	0.125 (0.0663 to 0.184)	<0.001
Estimated glomerular filtration rate/10 (ml/min/1.73 m^2^)	−0.0308 (−0.0628 to 0.0012)	0.059
White cell count (10^9^/L)	0.0234 (−0.0007 to 0.0476)	0.057
Number of medicines	0.0347 (0.0063 to 0.0630)	0.016
Previous allergy§	0.272 (0.0591 to 0.484)	0.012
Nervous system and mental disorders§	0.354 (0.0156 to 0.693)	0.040
Respiratory system§	−0.234 (−0.493 to 0.0253)	0.077
Gastrointestinal system§	−0.533 (−0.911 to −0.156)	0.006
Aminoglycosides and glycopeptides§	0.331 (−0.0457 to 0.708)	0.085
Other systemic antimicrobials§	0.311 (0.0777 to 0.545)	0.009
Epilepsy medicines§	0.385 (0.0950 to 0.675)	0.009
Constant	−1.674	

*Relationship between the independent and dependent variable (amount of increase in predicted log odds of the outcome event that would be predicted by a one unit increase in the independent variable).

†Original regression coefficients corrected by uniform linear shrinkage factor (0.855).

‡Test for difference between admissions with and without occurrence of outcome event. Obtained from multivariable regression modelling.

§Categorical exposure variable. For the purposes of calculating the predicted risk for individual patients, categorical variables are coded as ‘one’ if present and ‘zero’ if absent.

The c-index for the unadjusted model was 0.681, 95% CI 0.654 to 0.708, with good calibration (see online supplementary figure S3; calibration slope 0.974, intercept 0.012). Following bootstrapping, the c-index was 0.657; optimism for the calibration slope (0.855) was used as a linear shrinkage factor to adjust the regression coefficients of the regression model^56^ (table 2).

10.1136/bmjqs-2018-008335.supp3 Supplementary data Calibration plot of predicted probability of an outcome event against the proportion of admissions that experienced an event

The resulting regression equation can be used to calculate predicted risk for an individual patient (online supplementary appendix S4).

10.1136/bmjqs-2018-008335.supp4 Supplementary data Full regression equation for MOAT

The sensitivity of the MOAT at the decision threshold separating low-risk and medium-risk patients was 89.9% (95% CI 87.6% to 92.4%), specificity 30.2% (95% CI 27.2% to 33.2%) and positive predictive value (PPV) 46.8% (95% CI 45.5% to 48.1%). At the threshold between medium-risk and high-risk patients, the MOAT’s sensitivity was 66.2% (95% CI 62.4% to 70.0%), specificity 61.0% (95% CI 57.8% to 64.2%) and PPV 53.7% (95% CI 51.2% to 56.2%). These decision thresholds represent the minimum predicted risk probabilities that justify pharmacists’ input. As described in online supplementary appendix S1, the low/medium threshold was selected to identify 90% of patients likely to experience the study outcome; the medium/high threshold was informed by workload pressures, identifying the patients pharmacists should prioritise if they only have capacity to see 50%; it therefore provides a pragmatic indication of the potential clinical usefulness of the MOAT during periods of limited staffing.

**Figure 2:**
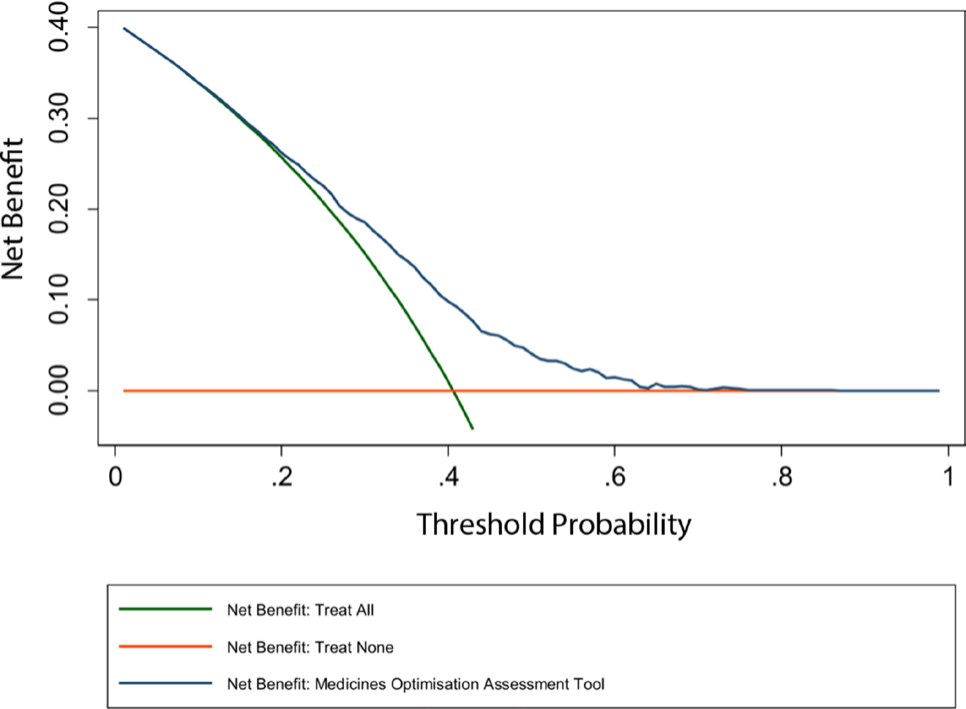
Decision curve for the Medicines Optimisation Assessment Tool.

The decision curve for the MOAT is shown in figure 2 and more detailed results in online supplementary appendix S2. In summary, between threshold probabilities of approximately 15% and 70%, the MOAT is better than both the ‘treat none’ and ‘treat all’ strategies, suggesting it is of value for threshold probabilities within this range.^48^ As both the low/medium and medium/high risk thresholds fall within this range (25% and 35% predicted risk probability, respectively), the MOAT can be considered to be clinically useful. Should a higher decision threshold be selected (due to extreme work pressures), the MOAT would continue to be of value in terms of clinical decision-making up to a threshold probability of approximately 70%, suggesting significant flexibility.

**Figure 3:**
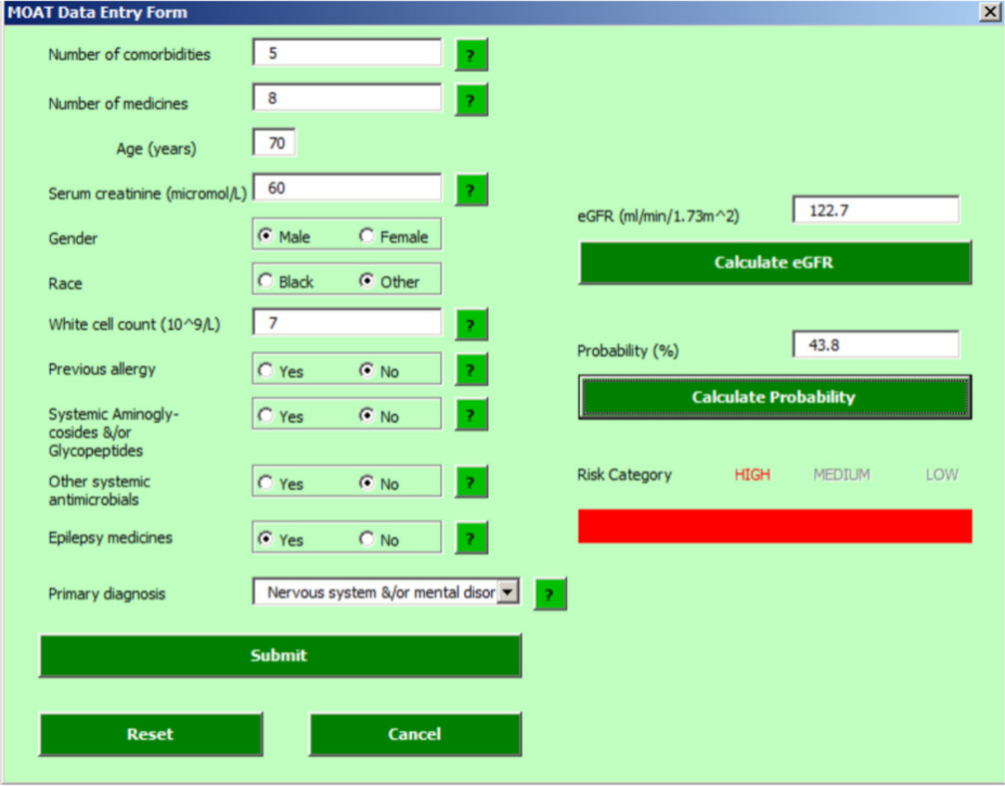
Screenshot of Medicines Optimisation Assessment Tool data entry sheet.

The resulting MOAT was developed as a Microsoft Excel sheet (figure 3), which calculates the estimated glomerular filtration rate, predicted probability of experiencing a moderate or severe preventable MRP and the patient’s risk category.

### Assessment of clinical credibility

The MOAT was perceived as clinically credible and usable by practising pharmacy professionals (online supplementary appendix S2). Additionally, the workload implications, based on the time taken to apply the MOAT compared with potential time saved by deprioritising low-risk patients, were considered to be reasonable. The results also suggest that ‘false negative’ patients may experience fewer outcome events that are of lower severity, compared with patients categorised as medium or high-risk.

## Discussion

### Key findings

Over 40% of admissions experienced an outcome event, namely at least one moderate or severe preventable MRP. The predictive performance of the MOAT was fair (c-index 0.66), with a sensitivity of 90% for the low/medium risk threshold (specificity 30%, PPV 47%) and 66% for the medium/high risk threshold (specificity 61%, PPV 54%). Decision curve analysis suggests that the MOAT has the potential to be clinically useful in guiding decision-making at these clinically relevant decision thresholds.

### Comparison with previous work

The MRP prevalence found in the present study (59.5%) is consistent with previous research. Blix *et al* 57 reported a prevalence of 81%; the data collection method and sample population were similar to the present study, but the higher prevalence may be explained by differences in MRP categorisation. Two more recent studies (both using similar methods to the present study), reported MRP prevalence rates of 52% and 53%.^58 59^ No previous estimate for the prevalence of *moderate or severe preventable* MRPs exists, but our results are consistent with Blix’s finding that approximately half of all MRPs experienced by 81% of hospitalised patients were ‘extremely important’ or ‘major’ in terms of clinical significance.^57^

Other statistical models to predict adverse medication-related outcomes in hospitalised patients have been developed,^16–22^ but it is not possible to make direct comparisons with the MOAT due to differences in the type of outcome predicted and/or their proposed target age group, with four models predicting risk in adults over 65 years only.^17 18 20 21^ Of these existing models, five are reported to have satisfactory predictive performance (c-index of 0.70–0.78),^16–18 21 22^ but all have methodological shortcomings and limitations that may limit their potential reliability and/or applicability, particularly those developed prior to publication of the Prognosis Research Strategy (PROGRESS) Partnership and TRIPOD recommendations.^28–30^ Methodological shortcomings include: (1) use of inadequate EPV, with only two of the five studies using an EPV of 10 or more;^16 17^ (2) poor reporting on quantity and handling of missing data (all studies); (3) use of univariable analysis to select predictors for inclusion in modelling (all studies); (4) categorisation of predictors during modelling^16–18 21^ and (5) no adjustment for overoptimistic predictions.^16–18 21^ Regarding potential limitations in use, risk groups were not created for three of the models^16 21 22^; one requires complex categorisation such as calculation of the Charlson index;^16^ and all use unclear predictor definitions. To our knowledge, the present study is also the first to use decision curve analysis to assess clinical usefulness.

Of five studies that developed prediction tools based on expert opinion,^23–27^ two provide no data on predictive performance.^23 26^ Of the remaining studies, it is difficult to make direct comparisons with the MOAT due to limitations such as small sample sizes,^27 60^ or lack of methodological information.^25^ There are also potential limitations related to the usability of existing tools, such as inclusion of predictors with subjective assessments,^24^ the need for fully integrated electronic information systems^24 25^ or complex predictor categorisation.^27^

In summary, we believe that none of the existing prediction tools have evidence for sufficient predictive accuracy and/or generalisability to recommend them for routine use outside of the site where they were developed. Although the MOAT’s c-index is slightly lower than some of the other prediction models reviewed, it has advantages in terms of robust methodology, which increases its potential reliability, usability and generalisability.

### Implications for practice

While it is not possible to advocate routine use of the MOAT prior to completion of external validation,^29^ the MOAT has potential to be applicable to adult hospitalised medical patients (general, acute and elderly medicine), irrespective of age. Given the diverse characteristics of the sample population (table 1), the MOAT also has potential applicability to a wide range of patients in terms of age, ethnicity, comorbidities and medical conditions. The MOAT was also perceived as clinically credible and usable by practising pharmacy professionals.

The MOAT has a modest c-index (0.66), but while the discriminative ability of a prognostic model is important, Steyerberg advises that ‘it is not possible to indicate a minimum value for the c-index to make a model clinically useful’.^38^ This is because the c-index alone does not consider the consequence of false positive or false negatives predictions.^38 49^ Use of decision thresholds, that is, the minimum predicted risk probabilities to justify pharmacists’ input, allowed calculation of the MOAT’s classification measures (sensitivity, specificity and predictive values). This permitted performance to be assessed at clinically relevant thresholds as opposed to the entire range of model-predicted probabilities.^61^ In terms of clinical utility, our aim was to produce a prediction tool able to correctly identify 90% of patients likely to experience a moderate or severe preventable MRP (ie, 90% sensitivity), a level of accuracy deemed appropriate by previous researchers^62^ and confirmed by our own research.^63^ We were able to achieve 90% sensitivity at the low/medium risk threshold (equivalent to a 25% predicted probability that a patient will experience an outcome event), meaning that only 10% of patients experienced the study outcome despite having a predicted probability below this threshold; our data also suggest these false negative patients experienced fewer outcome events that were of lower severity, compared with the true positive patients. While specificity at the low/medium risk threshold was modest (30%), the MOAT identified the 22% of patients least likely to experience the study outcome. We believe that the low/medium risk decision threshold is therefore reasonable in terms of the risks and benefits of the MOAT. These results also suggest that the MOAT has potential to prioritise pharmacists’ input while maintaining patient safety, a recognised need for clinical pharmacy services given limited resources and increasing demands.^64^

We were able to further investigate the potential clinical usefulness of the MOAT using decision curve analysis. Decision curves inform the range of threshold probabilities where prediction models would be of value in clinical practice, measured as the net benefit (a theoretical relationship between threshold probabilities and the relative value of false positive and negative results).^48^ As with classification measures, decision curves therefore permit assessment of performance at clinically relevant thresholds. The MOAT’s decision curve suggests net benefit across a significant range of threshold probabilities (15%–70%); decision thresholds within this range therefore have potential to be useful in clinical practice in guiding decision-making. As above, our aim was for 90% sensitivity, which was achieved with a decision threshold of 25% predicted risk probability (the low/medium risk threshold). This threshold, plus the medium/high risk threshold, used to indicate clinical usefulness during limited staffing (35% predicted risk probability), both fall within the range shown to have ‘net benefit’; the MOAT can therefore be considered to be clinically useful at thresholds that are relevant in practice. Furthermore, the creation of risk groups permits pharmacists to take account of workload capacity when prioritising patients, as does the reporting of both the predicted risk probability and risk group for individual patients.

The MOAT may require development of implementation strategies regarding the level of pharmacy input required by patients dependent on their risk categorisation. This might range from either no intervention for low-risk patients or one short face-to-face discussion following admission, to more intensive interventions for patients in higher risk categories (such as medicines reconciliation and medication review). It may also be possible to combine the MOAT with other triggers for pharmacy review, for example, swallowing difficulties, end of life care or risk of MRPs postdischarge; potentially the MOAT could then be used as part of a suite of tools, permitting prioritisation of patients and appropriate allocation of workload among team members based on skills and expertise. The development of these types of implementation strategies may also address patients’ views of safety; the medical view of patient safety often focusses on outcomes and avoidance of harm,^65^ whereas patients tend to focus on what makes them ‘feel safe’, including processes of care, and interpersonal dynamics with care providers.^66 67^ The MOAT inherently fits a medical view of safety, with attention on ‘risk reduction’; incorporation of the MOAT into a holistic system offering tailored input to patients may therefore help provide a sense of safety for all patients.

### Strengths and limitations

To the best of our knowledge, the MOAT is the first evidence-based clinical prioritisation tool to identify inpatients most in need of pharmacists’ input (in terms of their risk of moderate or severe preventable MRPs).

Strengths of this research include adherence to the PROGRESS^28 29^ and TRIPOD recommendations,^30^ at all stages of MOAT development. Other strengths include use of two study sites with markedly different patient demographics to increase generalisability, involvement of healthcare professionals and patient/public representatives in selection of the decision thresholds, creation of risk groups, use of decision curve analysis and development of an electronic decision aid to simplify use and indicate a course of action.^38 42^ The choice of predictors is also a potential strength of this research, as their perceived relevance and ease of use are crucial to the clinical credibility of prediction models.^68^ We chose predictors with data that are readily available in clinical practice (to avoid the need for additional measurements),^68^ and avoided the need for complex calculations or categorisation (to ensure ease of use).^68^ We also chose clear predictor definitions to ensure standardisation and reproducibility (to enhance generalisability and applicability of study results to practice).^69^

A limitation of the study is possible underestimation of the prevalence of MRPs due to pharmacists missing MRPs or not documenting them (as the MRP identification assessment exercise may suggest). This highlights the need for robust external validation of the MOAT, including the possible need for updating or recalibration.^42^ Another limitation was the inability to include predictors that are not routinely measured/recorded in clinical practice, have low prevalence or had potential measurement error, due to the potential for inaccurate results.^31 70 71^ While this may be appropriate,^38^ data on excluded predictors will need to be shared with MOAT users to inform implementation. The observational nature of the study is another potential limitation, as data collection was not carried out under strict trial conditions, although this did permit the MOAT to reflect clinical practice in terms of MRP identification. Finally, the presence of a small amount of missing predictor data, and subsequent use of multiple imputation may be a limitation, although as data appeared to be ‘missing at random’, multiple imputation was less likely to introduce selection bias than complete-case analysis,^42 72^ in addition to being statistically more efficient.^73^

### Implications for future research

External validation will be required to assess the MOAT’s accuracy and generalisability in a new group of patients.^29^ Following this, impact and implementation studies will be required to establish whether the MOAT has advantages over current practice, is compatible with (and can easily be incorporated into) practice, has the potential to change pharmacists’ behaviour, has a positive impact on patient outcomes and is cost effective.^50^

Further research may also be warranted into the use of risk categories. Organisations may differ in workforce capacity and/or aversion to risk, resulting in a need to develop flexible thresholds, tailored either to organisational need or fluctuating staffing levels.

Another potential future development for the MOAT includes integration into automated systems such as electronic health records systems. This could result in the ability to perform accurate, automated risk assessments in ‘real-time’, which would further support implementation into clinical environments. It may also be possible to assess the transportability of the MOAT to determine its ability to produce accurate predictions among people drawn from different but plausibly related populations,^74^ such as surgical patients, or patients in care homes.

## Conclusion

We have developed and internally validated a prognostic model to permit targeting of hospital patients most in need of pharmacists’ input based on their risk of moderate or severe preventable MRPs. Extensive external validation, involving prospective validation in a new cohort, will be required to further assess accuracy and generalisability before routine use can be recommended. Further research will also be required in terms of impact and implementation studies to assess the extent to which use of the MOAT may affect decision-making, improve efficiency or improve health outcomes.
