# Supplementary material for: Development and performance evaluation of the Medicines Optimisation Assessment Tool (MOAT): a prognostic model to target hospital pharmacists’ input to prevent medication-related problems
Source: BMJ Qual Saf. 2019 Mar 7;28(8):645–56. doi: 10.1136/bmjqs-2018-008335 (PMC6716361; doi:10.1136/bmjqs-2018-008335)
Supplement: Supplementary data [file bmjqs-2018-008335supp001.pdf]

S1 – Supplementary information on methods

OUTCOME CATEGORISATION (PREVENTABILITY AND SEVERITY)

Preventability

Pharmacy staff at the study sites received training on the identification and recording of medication related problems (MRPs) prior to commencement of the study, including how to assess for preventability. Medication errors, a significant subset of MRPs, are defined as 'a preventable event that may cause or lead to inappropriate medication use or patient harm while the medication is in the control of the health care professional, patient, or consumer. Such events may be related to professional practice, health care products, procedures, and systems, including prescribing, order communication, product labeling, packaging, and nomenclature, compounding, dispensing, distribution, administration, education, monitoring, and use'.[1] Staff were therefore advised to apply this definition to all MPRs identified, and to classify all MRPs that met the definition as preventable.

Any MRPs that did not meet this definition, for example adverse drug reactions, where therefore classed as non-preventable. Examples of non-preventable MRPs include hallucinations in a patient prescribed zopiclone, and bronchospasm in a patient prescribed atenolol.

Severity

As described in a published protocol,[2] each MRP was assessed by an expert panel and agreement reached by consensus on whether it was a true MRP (expressed as a dichotomous variable of yes or no). Confirmed MRPs that were considered to be preventable (as described above), were then assessed for severity using a visual analogue scale.[3] This method requires four experienced healthcare professionals (pharmacists, medical, or nursing staff) to independently score each event in terms of potential patient outcomes on a scale of zero to ten. Zero represents a case with no potential adverse effect on the patient, and ten a case that would result in death. The mean score for each event is then used as an index of severity, with a score of less than three being considered as a minor outcome (very unlikely to have an adverse effect), a score of three to seven considered as moderate (likely to cause some adverse effects or interfere with therapeutic goals, but very unlikely to result in death or lasting impairment), and a score of greater than seven considered to be a severe outcome (likely to cause death or lasting impairment).

Examples of minor outcomes included selection errors by prescribers (where dispersible tablets prescribed when standard tablets intended), non-intentional omission of non-critical medicines on admission (for example laxatives), and inappropriate timing of administration by prescribers (for example loop diuretics prescribed in the evening, thereby potentially interfering with sleep).

Moderate outcomes included inadequate therapeutic drug monitoring (such as for intravenous gentamicin), significant drug-drug interactions (for example concomitant use of simvastatin and clarithromycin), and use of inappropriate drug doses (for example failure to reduce the dose of apixaban to take account of renal impairment).

There was one outcome graded as severe. This involved a patient prescribed prophylactic heparin (for thromboprophylaxis) despite having a raised international normalised ratio (of 7.9), leading to increased risk of bleeding.

SELECTION OF CANDIDATE PREDICTORS

A summary of the methods used to operationalise methodological recommendations for the selection of candidate predictors is given in Table S1.1.

Table S1.1 – Selection recommendations for candidate predictors and method of operationalisation

Methodological recommendation	Method of operationalisation
Predictors reported as prognostic should be included[4 5]	Literature review performed and simple count used to identify predictors.<br>Strength of evidence categorised into tertiles, i.e. 'low' if association found in 33% or fewer studies (that investigated the predictor), 'moderate' if higher than 33% but fewer than 66%, and 'high' if higher than 66%.
Selection of predictors should be informed by clinical understanding[5 6]	Expert survey of healthcare professionals and patient/public representatives to obtain clinical understanding and lay views on potential predictors.
If predictors are highly correlated only one should be selected[5]	Where high interdependency anticipated, one predictor excluded based on level of evidence/other methodological recommendations.
Predictors that occur infrequently can lead to inaccurate results[5 7]	Predictor excluded if estimated occurrence <10% patients.*
Predictors should be available at the time model intended to be used[8]	Predictor excluded if data not available on day patient admitted to hospital.

Continued from previous page…

Methodological recommendation	Method of operationalisation
Predictors should be clearly defined, standardised, and reproducible[8]	Predictor excluded if definition subjective and/or subjective measurement scale used.
Predictors should have minimal measurement error[9]	Predictor excluded if low reliability anticipated with test-retest, intra-rater and/or inter-rater measurements.
Predictors should form part of standard clinical datasets	Predictor excluded if not included in standard medical records and/or estimated data availability <50% patients.†

* 5% used in previous prognostic model studies,[10-12] but 10% selected to allow for estimation error (as review based on personal clinical experience/knowledge)

† Based on Steyerberg's recommendation that predictors with more than 50% missing data generally mistrusted[13]

As described in a published protocol,[2] a review of the published literature identified 59 possible predictors, but substantial variations were found between studies in terms of the strength of evidence for each predictor. This is potentially due to significant differences in study design, and the outcome measure used (namely adverse drug events, adverse drug reactions, prescribing errors and MRPs). Twenty seven of the potential predictors were selected, based on an assessment of suitablilty in terms of methodological requirements, for inclusion in a survey to obtain expert opinion from healthcare professionals and patient/public representatives. The survey was administered during April-June 2016, and a total of 247 responses received. The results showed that the majority of the potential predictors (23 of 27) were considered 'important' or 'very important'. In addition, a significant number of additional predictors (59) were suggested.[14] The final selection of candidate predictors was made following an assessment of the potential predictors included in the expert survey and additional predictors suggested by survey respondents. Each predictor was assessed against the recommendations for candidate predictors: the strength of evidence from previous research (where available), clinical understanding and lay views (in terms of survey scores for perceived importance/clinical relevance, or number of times additional predictors suggested by respondents), and the remaining methodological recommendations for candidate predictors as summarised in Table S1.1.

Details of all predictors considered, with reasons for inclusion/exclusion, are summarised below.

Predictors excluded from expert survey

The 32 predictors that were considered for inclusion in the expert survey, but not selected, are summarised in Table S1.2, with reason(s).

Table S1.2 – Potential predictors excluded from the expert survey with reason(s)

Predictor	Reason(s) for non-selection						
	High correlation with other predictor(s)	Occurs infrequently*	Not available when model intended to be used	Not clearly defined / subjective definition / not reproducible	Potential measurement error	Not part of standard datasets &/or not reliably recorded†	Other (see details)
Non-native speaker				✓	✓	✓	
Marital status							Unlikely to be associated with MRPs during hospital stay
Weight/height related factors						✓	
Non-compliance with medication				✓	✓	✓	
Disability				✓	✓	✓	
Ability to sign consent form				✓	✓	✓	
Smoking status/nicotine use				✓	✓	✓	
Alcohol related				✓	✓	✓	
Falls risk				✓	✓	✓	
Impaired manual skills				✓	✓	✓	
Visual impairment				✓	✓	✓	
Cessation of medicines used before admission				✓	✓	✓	
Prescription of new medicines during/before admission				✓	✓	✓	
Length of stay			✓				
Time of day prescribed						✓	Not patient specific
Month of stay							Not patient specific
Stage of patient stay (admission/ during stay/discharge)							MOAT not intended to target stage of pharmacist input
Comorbidity index	✓					✓	Number of comorbidities included
DRG weight	✓					✓	Diagnostic categories included
Anaemia/haemoglobin	✓						
Temperature	✓						
Heart rate/blood pressure	✓						
Serum amylase						✓	
Thyroid function						✓	
Serum calcium	✓						
Prothrombin time/INR	✓						
Blood glucose/HbA1c	✓					✓	
Serum C-reactive protein	✓						
‘ISMP high-alert medication’/ risk of harm							Alternative method used to categorise high-risk medicine use
‘Narrow therapeutic index’ medicines							Alternative method used to categorise high-risk medicine use
Drug interactions				✓	✓		
Drug dose (high versus low)				✓	✓		

* Estimated occurrence <10%

† Not included in in standard medical records and/or estimated that data available for <50% patients

DRG = Diagnosis-related group, INR = International Normalised Ratio, HbA1c = Haemoglobin A1c/glycated haemoglobin test, ISMP = Institute for Safe Medication Practices

Results of expert survey

The review of predictors included in the expert survey is summarised in Table S1.3, with reasons for inclusion/exclusion as candidate predictors.

Table S1.3 – Review of candidate predictors included in expert survey

Predictor	Strength of published evidence*	Survey results		Low correlation with other predictor(s)	Estimated occurrence ≥ 10%	Available when model intended to be used	Clearly defined / reproducible	Minimal measurement error	Part of standard clinical datasets / reliably recorded‡	Selected as a candidate predictor
		Median response†	Interquartile range							
Renal function	High	1	0	✓	✓	✓	✓	✓	✓	✓
Liver function	Mod	1	1	✓	✓	✓	✓	✓	✓	✓
Age	Mod	1	1	✓	✓	✓	✓	✓	✓	✓
Comorbidities	High	1	1	✓	✓	✓	✓	✓	✓	✓
Allergies	High	1	1	✓	✓	✓	✓	✓	✓	✓
Swallowing problems	Mod	1	1	✓	✓	✓	x	x	x	x
Number of medicines prescribed	High	1	1	✓	✓	✓	✓	✓	✓	✓
Number of potentially inappropriate medicines prescribed	High	1	1	✓	✓	✓	✓	✓	x	x
Type of medicine prescribed	High	1.5	1	✓	✓	✓	✓	✓	✓	✓
Serum sodium level	High	2	1	✓	✓	✓	✓	✓	✓	✓
Serum potassium level	High	2	1	✓	✓	✓	✓	✓	✓	✓
Platelet count	High	2	1	✓	✓	✓	✓	✓	✓	✓
Serum albumin level	Mod	2	1	✓	✓	✓	✓	✓	✓	✓
White blood cell count	High	2	2	✓	✓	✓	✓	✓	✓	✓
Diagnosis/reason for admission	Mod	2	1	✓	✓	✓	✓	✓	✓	✓
Type of hospital department/speciality	High	2	1	x	✓	✓	✓	✓	✓	x
Readmission to hospital within 30 days	Mod	2	1	x	✓	✓	✓	✓	x	x
Number of hospital admissions within 6 months	Low	2	1	✓	✓	✓	✓	✓	✓	✓
Elective versus unplanned admission	Mod	2	1	✓	x	✓	✓	✓	✓	x
Route of administration of medication	High	2	1	✓	✓	✓	✓	✓	✓	✓
Dosing frequency of medication	High	2	1	x	✓	✓	✓	✓	✓	x
Social deprivation	Low	2	1	✓	✓	✓	✓	✓	x	✓§
Dependent living situation	Low	2	1	✓	✓	✓	x	x	x	x
Ethnicity	Low	3	2	✓	✓	✓	✓	✓	✓	x
Hyperlipidaemia	Mod	3	2	✓	✓	✓	✓	✓	x	x
Number of outpatient appointments within 6 months	Low	3	1	x	✓	✓	✓	✓	✓	x
Gender	Low	4	1	✓	✓	✓	✓	✓	✓	x

* Strength of evidence categorised as 'Low' association found in ≤33% published studies, 'Mod' (moderate) >33% and <66%, 'High' ≥66%

† Likert responses allocated ordinal numbers, 1= very important, 2=important, 3=50:50, 4=less important, 5=not important

‡ Included in standard admission proforma at study sites and/or data available for ≥50% patients (based on a review of the patient records of 84 patients, 50 from Hospital A and 34 from Hospital B)

§ Not routinely recorded in medical records, but can be calculated from postcode

Additional predictors suggested by survey respondents

The review of additional predictors suggested by survey respondents is summarised in Table S1.4. Two of the 59 predictors, dementia and weight, were selected as candidate predictors. Dementia was included as dementia/cognitive function received a high number of suggestions (34), and dementia meets the remaining methodological requirements. We had previously excluded weight based on estimated data availability, but further assessment found that it would be possible to calculate the body mass index for 62 of 84 patients reviewed (74%) therefore this was also selected as a candidate predictor.

Table S1.4 – Review of candidate predictors suggested by survey respondents

Predictor	Reason(s) for non-selection							
	High correlation with other predictor(s)	Occurs infrequently*	Not available when model intended to be used	Not clearly defined / subjective definition / not reproducible	Potential measurement error	Not part of standard datasets &/or not reliably recorded†	Related to problems encountered by patients in primary care	Other / comments
Over the counter/herbal medicine use				✓	✓	✓		
Length of time on medicine/newly prescribed				✓	✓	✓		
Medication Regimen Complexity Index						✓	✓	
Irregular dose and administration				✓	✓		✓	
Anticholinergic burden						✓		
Medicine use ‘off label’						✓		
Homecare provided medicines		✓					✓	
Length and appropriateness of antibiotic treatment								Antibiotics analysed as high-risk medicines
Use of an antidote e.g. naloxone, vitamin K								Sign of MRP rather than predictor
Constituents in formulations that may be pharmacologically active	✓							
Medicines or combination of medicines that predispose falls				✓		✓	✓	
Dementia								INCLUDED
Cognitive function/mental capacity/mental health status/confusion/delirium				✓	✓	✓		
Adherence/compliance				✓		✓	✓	
Physical/sensory impairment				✓		✓	✓	
Patient health beliefs/behaviours				✓		✓	✓	

Continued from previous page…

Predictor	Reason(s) for non-selection							Other / comments
	High correlation with other predictor(s)	Occurs infrequently*	Not available when model intended to be used	Not clearly defined / subjective definition / not reproducible	Potential measurement error	Not part of standard datasets &/or not reliably recorded†	Related to problems encountered by patients in primary care	
Compliance aid						✓	✓	
Frailty score						✓	✓	
Language barrier				✓		✓	✓	
Self-care for medicines/whether patient/family/carer is responsible for medicines				✓		✓	✓	
Carer status				✓		✓	✓	
Intellectual disability/learning difficulty		✓		✓		✓	✓	
Poor health literacy				✓		✓	✓	
Weight (obese and anorexia)								INCLUDED
Patient education level/literacy				✓		✓	✓	
Recreational drugs/substance misuse		✓		✓		✓	✓	
End of life care		✓						
Patient/carer level of knowledge/patient baseline understanding of disease state/medication				✓		✓	✓	
Alcohol use/misuse				✓			✓	
Nil by mouth/enteral tube						✓		
Social/cultural issues				✓		✓	✓	
Overdose risk/previous overdose/misuse of medication				✓		✓	✓	
Falls risk				✓		✓	✓	
Housing status (e.g. homeless)		✓		✓		✓	✓	
Activities of Daily Living score/functional level						✓	✓	
Medicines related admission				✓		✓		
Social-related admission		✓		✓		✓	✓	
Capacity as defined by Mental Capacity Act		✓		✓		✓	✓	
Member of travelling community		✓				✓		
Elderly living alone							✓	
Decanting of medicines occurring				✓			✓	
Significant weight changes				✓		✓	✓	
Bariatric patient		✓				✓		
Nutritional status				✓				
Smoking status								Low strength of evidence
Transgender		✓		✓				
Housebound						✓		
Disability				✓		✓	✓	
Pain score				✓			✓	
Venous access patient/type of cannula								Cannulas routinely used for all study patients
Having received antibiotics in last 3 months						✓		
Frequency of GP contact	✓					✓		
Identifying if patient is on a risk register with general practitioner		✓				✓	✓	
Pregnancy/breastfeeding		✓						

Continued from previous page…

Predictor	Reason(s) for non-selection							
	High correlation with other predictor(s)	Occurs infrequently*	Not available when model intended to be used	Not clearly defined / subjective definition / not reproducible	Potential measurement error	Not part of standard datasets &/or not reliably recorded†	Related to problems encountered by patients in primary care	Other / comments
Requirement to manipulate the medicine before administration				✓				
Critical care admission		✓						
Staffing levels on ward/hospital				✓		✓		Not patient specific
Communication problems across interfaces				✓		✓		
The days admitted/time of year								Not patient specific
Number of patient transfers across wards						✓		

* Estimated occurrence <10% patients

† Not included in standard admission proforma at study sites and/or data available for <50% patients (based on a review of the patient records of 84 patients, 50 from Hospital A and 34 from Hospital B)

MRP = medication related problem

MRP IDENTIFICATION ASSESSMENT EXERCISE

MRP data for this study were collected by pharmacy staff at the study sites as part of their routine daily clinical assessment of patients. A potential limitation was the possible impact of knowledge, experience and skills of pharmacists on their ability to identify potential MRPs. The aim of the MRP identification assessment exercise was therefore to quantify potential variability in MRP identification by pharmacists.

All pharmacy staff involved in the study were asked to complete the MRP identification assessment exercise. The assessment was completed anonymously and no time limit was set. Pharmacists were instructed to review each medication chart and list the potential MRPs identified using a form designed for this purpose. They were not told the number of potential MRPs on each medication chart, or the overall number. Pharmacists were permitted to refer to references sources if required (to reflect standard clinical practice).

MISSING DATA

It is known that differences in the extent and type of missing data, and the methods used to handle this missing data, may greatly influence model development and predictive performance of prognostic models.[9]

To assess the potential impact of missing candidate predictor data we calculated the number of admissions with missing data, number of values missing, and number of missing values for each variable. We then compared characteristics for admissions with missing values and those with completely observed data to inform possible reasons for the missingness.[15] This was on the basis that where data are ‘missing completely at random’, it is considered reasonable to use complete-case analysis, as participants with missing data are likely to be a truly random subset of the study sample.[9] Where missing data are related to other observed participant data, known as ‘missing at random’ (MAR), a complete-case analysis would lead to a non-random subset, and biased results.[9 16] Multiple imputation is generally considered to be the preferred method to handle data that are MAR,[9 16] but is not appropriate where data are ‘missing not at random’ (MNAR), as MNAR data are related to unobserved data, and therefore cannot be plausibly estimated from the observed study variables.

Missing candidate predictor data were handled using ‘multiple imputation by chained equations’ to create 30 imputed datasets (Stata version 14.2).[17 18] As it is not possible to distinguish between MAR and MNAR using observed data, sensitivity analyses were performed to investigate possible departure from the MAR assumption.[15 16] This involved comparing the multivariable logistic regression estimates for the complete-case and imputed datasets, with plausible explanations for the observed differences supporting the MAR assumption.[19]

TRUNCATION OF OUTLIERS

Outliers, defined by Tukey as values more than (or equal to) one and a half interquartile ranges (IQRs) above the third quartile or below the first quartile,[20] have the potential to substantially distort statistical estimates and inflate error rates.[21] As advised by Steyerberg,[13] ‘truncation’ was used to reduce the influence of outliers on the regression coefficients (known as leverage). This involved assessing the impact of shifting very high and very low values to truncation points, then examining the predictor-outcome relationship by comparing univariable logistic regressions coefficients for models specified with non-truncated and truncated data.

MODEL DEVELOPMENT

A random effects model was used to account for possible correlation between patients admitted more than once during the study period. Failure to take account of this lack of independence may have resulted in standard errors and *p* values being too small, hence

confidence intervals too narrow, resulting in a belief that evidence was stronger than it actually is.[22]

Univariable associations between predictors and the outcome event were not used to preselect variables; this is not recommended on the basis that important predictors may be excluded due to their predictive effect being masked by other predictors.[15] There is no consensus on the best method to select predictors during modelling, but forward selection has been shown to be less reliable than a backwards elimination approach;[9] we therefore chose to use backwards elimination. Backward elimination starts with a full model (i.e. one that contains all predictors), the 'least significant' predictor (based on a predetermined 'stopping rule'), is then removed, and the model re-fitted. This is continued until all predictors in the model are 'significant'.[13] We chose a significance level of p greater than 0.157 to exclude predictors, chosen as it is comparable with the more complex Akaike Information Criterion (AIC),[13] which compares models based on their fit to the data while penalising for the complexity of the model. Use of the AIC, or even higher p values (for example a p value of greater than 0.5), is considered to be a suitable for relatively small dataset (hence relatively larger p-values for the predictors) as this is less likely to result in underfitting than alternative methods.[13] Automated variable selection was not used because: (1) manual selection permitted clinical judgement to be incorporated, for example to decide which predictor to exclude in cases where two predictors had similar significance levels; and (2) automated techniques are generally considered to have a high probability of generating spurious findings.[5]

Model diagnostics included a check of the accuracy of the quadrature approximation,[23] evidence for specification error,[24] and an assessment of the impact of outlying observations;[24 25] no model adjustments were required.

INTERNAL VALIDATION

The predictive performance of prognostic models is overestimated when assessed using the same data used in development (known as the apparent performance), simply because the model has been optimised for that data. This results in overconfident predictions in independent data, where higher predictions are too high, and low predictions too low.[26] It is therefore recommended that all model development studies include some form of internal validation; for example split-sample validation (where the development data is divided into two datasets, one for model development and one for validation), or bootstrapping (which mimics the process of sampling from the underlying population by drawing random samples from the developmental dataset).[13 15] Bootstrap validation is generally regarded as the

preferred method as it permits all data to be used for model development, so is more statistically efficient.[9] In addition, bootstrap validation permits optimism to be quantified, and provides an estimate of any adjustments required.[13 15] Steyerberg advises that 100-200 bootstraps may be sufficient;[13] we chose to use 200 to increase the stability of the estimates. Each bootstrap sample was equivalent in size to the original dataset and drawn with replacement, meaning patients were included a number of times or not at all. We constructed a model in each of the bootstrap samples using similar steps to those used for the original regression model, then recorded the 'apparent performance' in terms of the concordance index (c-index) and calibration slope. Each bootstrap model was then applied to the original dataset and the performance (known as the 'test performance') recorded. The optimism was calculated as the average difference between the apparent and test performance of the bootstrap models. Bootstrapping suggested slight overfitting, the original model's c-index was therefore adjusted by subtracting the average optimism for the c-index. The average optimism for the calibration slope was also used as a 'linear shrinkage factor' to account for overconfident predictions; this involved multiplying each of the regression coefficients by the shrinkage factor.

CREATION OF RISK GROUPS

No consensus exists on how risk groups should be created for prognostic models,[15] but it is recommended that subject matter input is used rather than reliance on statistical estimation.[13] We chose to create three risk categories: high, medium and low, with the choice of cut-offs for the predicted risk probabilities (also known as decision thresholds)[13 27] guided by the target sensitivity of the Medicines Optimisation Assessment Tool (MOAT), and consensus views of practising pharmacy staff.

A decision threshold is the minimum predicted risk probabilities to justify an intervention, in this case, pharmacists' input. The decision threshold to distinguish between low and medium-risk patients was primarily informed by the MOAT's target sensitivity. An acceptable target sensitivity was established via a survey of healthcare professionals and patient/public representatives, administered during April-June 2016. Invitations to participate were shared through various fora/networks, including the Royal Pharmaceutical Society of Great Britain Research and Evaluation Network and Medication Safety Officers Network for England. It was also emailed directly to key individuals, for example the Medication Safety Team at NHS England, and researchers in the field (identified during literature review). All respondents were also requested to share the survey further within their networks/organisations. We proposed a target sensitivity of 90% (based on previous research to develop a 'clinical decision rule' for pharmacist prioritisation),[28] and asked survey respondents whether this

was acceptable. Of the 237 responses, 189 (80%) answered that 90% was an acceptable target; 21 (9%) answered no, and 27 (11%) were ‘unsure’. The professional role of the 237 respondents is summarised in Table S1.5.

Table S1.5 – Current role of survey respondents

Current role	Number	Percentage
Pharmacist/member of the pharmacy team	178	75.2
Doctor	31	13.1
Nurse	10	4.2
Academic (no other professional role stated)	10	4.2
Patient or public representative	6	2.5
Other healthcare professional	2	0.8

We used the nominal group technique (NGT) to confirm the acceptability of this decision threshold as part of a wider assessment of the MOAT’s clinical credibility.[29 30] Practising pharmacists and clinical pharmacy technicians from Hospital A were invited to volunteer and seven participants were included in the nominal group (as this has been reported as the maximum recommended number).[31] Pharmacy staff were chosen for this assessment as our aim was to obtain the views of clinicians who would ultimately use the MOAT in clinical practice. The nominal group comprised four clinical pharmacists and three clinical pharmacy technicians. This included two newly qualified pharmacists, one mid-grade specialist pharmacist, and one senior specialist pharmacist. The technicians were all experienced senior technicians. A standard NGT method was used,[31 32] comprising two meetings during which panellists discussed the issues, rated the questions posed (using a nine-point Likert scale), and then rerated the questions following further discussion. The group considered the sensitivity versus specificity of alternative decision thresholds, and the balance between false negative predictions and the number of patients requiring pharmacist review. There was consensus that a decision threshold corresponding to 90% sensitivity was appropriate. The group also agreed on a higher decision threshold to distinguish between medium and high-risk patients, informed by considering workload pressures. This threshold is equivalent to the point that separates patients into two equally sized risk groups, in other words, it identifies which patients pharmacists should prioritise if they are only able to see 50% of patients; while this threshold was selected arbitrarily, in that it does not consider the risks and benefit of use, it was intended to give a pragmatic indication of the potential clinical usefulness of the MOAT during periods of limited staffing.

The decision threshold to distinguish between low and medium-risk patients corresponds to a predicted risk probability of approximately 25% (further explanation of predicted probabilities given in appendix S4). Patients below this threshold were therefore categorised as low-risk. Given the 90% sensitivity of the MOAT at this decision threshold, only 10% of patients experienced a study outcome despite having a predicted probably below this threshold. A decision threshold to distinguish between medium and high-risk patients corresponds to a predicted risk probability of approximately 35%, with patients above this threshold categorised as high-risk. Patients were categorised as medium-risk if their predicted risk probabilities were between these two thresholds.

DECISION CURVE ANALYSIS

Once decision thresholds were selected, it was possible to assess clinical usefulness, in terms of whether the MOAT is likely to be beneficial in clinical practice for guiding decision making.[13] This goes beyond calculation of the c-index (which is primarily interested in predictive accuracy) to incorporate information on consequences, for example considering the relative impact of false negative and false positive predictions.[33]

Decision curve analysis has been suggested as a method to assess clinical usefulness.[13 34] This permits performance of a model to be assessed over a range of decision thresholds, using the theoretical relationship between threshold probabilities and the relative value of false positive and false negative results,[33] calculated as the net benefit:

$$Net\ benefit = \frac{true\ positive\ count}{total\ number\ of\ patients} - \frac{false\ postive\ count}{total\ number\ of\ patients} \times \left(\frac{threshold\ probability}{1 - threshold\ probability}\right)$$

Net benefit is interpreted in units of the true positives, and is a measure of how many more patients are correctly ‘treated’ (true positives) at the same rate of ‘not treating’ those who do not need treatment (false positives).[13]

By varying the threshold probability it is possible to produce a ‘decision curve’, with threshold probability plotted on the x-axis, and net benefit plotted on the y-axis. The net benefit of default policies of ‘treat none’ and ‘treat all’ are also plotted to permit comparisons to be made. The net benefit of ‘treating none’ is zero (as the true and false positive counts are both zero), therefore if the net benefit of the prediction model is positive, it is better to use the model than ‘treat none’. The true and false positive counts for the ‘treat all’ strategy are the number of patients with and without the outcome respectively; the net benefit of ‘treat all’ is therefore equal to the outcome prevalence at a threshold probability of zero, and equal to zero at the prevalence of the outcome.[33]

The decision curve informs the range of threshold probabilities for which the prediction model would be of value in clinical practice.[35] To interpret a decision curve one identifies a range of plausible threshold probabilities, and then determines whether the model has benefits (i.e. a net benefit greater than ‘treating all’ and ‘treating none’) at all values within this range.[13] We therefore plotted the decision curve for the MOAT to establish whether it has the potential to be clinically useful at the decision thresholds selected to distinguish between low, medium and high-risk patients.

ASSESSMENT OF CLINICAL CREDIBILITY

Consensus views on the MOAT

The NGT was used (as described above for the creation of risk groups). The panellists were asked to consider the following questions:

- does the MOAT demonstrate content validity? That is to say, would most clinicians consider that the choice of predictors is appropriate for the purpose of the prediction tool, that no obvious predictors are missing, and that individual predictors are appropriately grouped?;
- is the visual presentation of the MOAT reasonable?;
- does the MOAT have the potential to be ‘usable’ in clinical practice (related to simplicity of interpretation and time taken to apply the MOAT)?

Participants were asked to rate their responses using a nine-point Likert scale; scores of 1-3 represented disagreement, 4-6 an equivocal response, and 7-9 represented agreement.

Workload implications

Workload implications were assessed by:

- analysing the original MOAT developmental dataset to identify the proportion of patients who ‘screened positive’ (i.e. patients in the medium or high-risk groups), meaning they would require pharmacist review;
- calculating the median time required to apply the MOAT per patient.

For the second part of this assessment, four volunteers reviewed five patients each and recorded the time taken to use the MOAT (i.e. to obtain the required data and calculate the risk probability for each patient).

Clinical implication of false negative predictions

The outcome events (moderate or severe preventable MRPs) experienced by patients who were 'true positives' were compared with those experienced by patients who were 'false negatives'. The Mann-Whitney U test was used to test for differences in the severity scores and number of events experienced by both groups.

REFERENCES

1. Medication Errors: Technical Series on Safer Primary Care. Licence: CC BY-NC-SA 3.0 IGO ed: Geneva: World Health Organization, 2016.
2. Geeson C, Wei L, Franklin BD. Medicines Optimisation Assessment Tool (MOAT): a prognostic model to target hospital pharmacists' input to improve patient outcomes. Protocol for an observational study. *BMJ Open* 2017;7(6) doi: 10.1136/bmjopen-2017-017509
3. Dean BS, Barber ND. A validated, reliable method of scoring the severity of medication errors. *American Journal of Health-System Pharmacy* 1999;56(1):57-62.
4. Royston P, Moons KG, Altman DG, et al. Prognosis and prognostic research: developing a prognostic model. *Bmj* 2009;338:b604.
5. Katz MH. Multivariable analysis: a primer for readers of medical research. *Annals of internal medicine* 2003;138(8):644-50.
6. Bouwmeester W, Zuithoff NP, Mallett S, et al. Reporting and methods in clinical prediction research: a systematic review. *PLoS Med* 2012;9(5):e1001221.
7. Vittinghoff E, McCulloch CE. Relaxing the rule of ten events per variable in logistic and Cox regression. *American journal of epidemiology* 2007;165(6):710-18.
8. Moons KG, Royston P, Vergouwe Y, et al. Prognosis and prognostic research: what, why, and how? *Bmj* 2009;338:b375.
9. Moons KG, de Groot JA, Bouwmeester W, et al. Critical appraisal and data extraction for systematic reviews of prediction modelling studies: the CHARMS checklist. *PLoS Med* 2014;11(10):e1001744.
10. McElnay J, McCallion C, Al-Deagi F, et al. Development of a risk model for adverse drug events in the elderly. *Clinical drug investigation* 1997;13(1):47-55.
11. Trivalle C, Burlaud A, Ducimetière P, et al. Risk factors for adverse drug events in hospitalized elderly patients: a geriatric score. *European Geriatric Medicine* 2011;2(5):284-89.
12. Tangiisuran B, Scutt G, Stevenson J, et al. Development and validation of a risk model for predicting adverse drug reactions in older people during hospital stay: Brighton Adverse Drug Reactions Risk (BADRI) model. *PloS one* 2014;9(10):e111254.
13. Steyerberg E. Clinical prediction models : a practical approach to development, validation and updating: Springer 2009.
14. Geeson C, Franklin BD, Wei L. Identification of risk (prognostic) factors for medication related problems (MRPs) occurring during hospital admission: a survey of healthcare professionals and patient/public representatives. *International Journal of Pharmacy Practice* 2017;25:49-50. doi: 10.1111/ijpp.12368
15. Moons KG, Altman DG, Reitsma JB, et al. Transparent Reporting of a multivariable prediction model for Individual Prognosis Or Diagnosis (TRIPOD): Explanation and ElaborationThe TRIPOD Statement: Explanation and Elaboration. *Annals of internal medicine* 2015;162(1):W1-W73.
16. Vandenbroucke JP, Von Elm E, Altman DG, et al. Strengthening the Reporting of Observational Studies in Epidemiology (STROBE): explanation and elaboration. *PLoS Med* 2007;4(10):e297.

17. StataCorp. STATA MULTIPLE-IMPUTATION, REFERENCE MANUAL, RELEASE 13: Stata Press; [Available from: https://www.stata.com/manuals13/mi.pdf accessed September 2018.
18. Sterne JA, White IR, Carlin JB, et al. Multiple imputation for missing data in epidemiological and clinical research: potential and pitfalls. *Bmj* 2009;338:b2393.
19. Marchenko YV, Eddings W. A note on how to perform multiple-imputation diagnostics in Stata. *College Station, TX: StataCorp* 2011
20. Hoaglin DC, Iglewicz B, Tukey JW. Performance of some resistant rules for outlier labeling. *Journal of the American Statistical Association* 1986;81(396):991-99.
21. Osborne JW, Overbay A. The power of outliers (and why researchers should always check for them). *Practical assessment, research & evaluation* 2004;9(6):1-12.
22. Kirkwood BR, Sterne JA. Essential medical statistics: John Wiley & Sons 2010.
23. StataCorp. quadchk — Check sensitivity of quadrature approximation: Stata Press; [Available from: https://www.stata.com/manuals13/xtquadchk.pdf accessed September 2018.
24. UCLA. LESSON 3 LOGISTIC REGRESSION DIAGNOSTICS: UCLA: Statistical Consulting Group; [Available from: https://stats.idre.ucla.edu/stata/webbooks/logistic/chapter3/lesson-3-logistic-regression-diagnostics-2/ accessed September 2018.
25. Sarkar SK, Midi H, Rana S. Detection of outliers and influential observations in binary logistic regression: An empirical study. *Journal of Applied Sciences* 2011;11:26-35.
26. Steyerberg E, Eijkemans M, Habbema J. Application of shrinkage techniques in logistic regression analysis: a case study. *Statistica Neerlandica* 2001;55(1):76-88.
27. Steyerberg EW, Vergouwe Y. Towards better clinical prediction models: seven steps for development and an ABCD for validation. *European heart journal* 2014;35(29):1925-31.
28. Hohl CM, Yu E, Hunte GS, et al. Clinical Decision Rules to Improve the Detection of Adverse Drug Events in Emergency Department Patients. *Academic Emergency Medicine* 2012;19(6):640-49. doi: 10.1111/j.1553-2712.2012.01379.x
29. Stiell IG, Wells GA. Methodologic standards for the development of clinical decision rules in emergency medicine. *Annals of emergency medicine* 1999;33(4):437-47.
30. Laupacis A, Sekar N. Clinical prediction rules: a review and suggested modifications of methodological standards. *Jama* 1997;277(6):488-94.
31. McMillan SS, King M, Tully MP. How to use the nominal group and Delphi techniques. *International journal of clinical pharmacy* 2016;38(3):655-62.
32. Jones J, Hunter D. Consensus methods for medical and health services research. *BMJ: British Medical Journal* 1995;311(7001):376.
33. Vickers AJ, Elkin EB. Decision curve analysis: a novel method for evaluating prediction models. *Medical Decision Making* 2006;26(6):565-74.
34. Steyerberg EW, Vickers AJ, Cook NR, et al. Assessing the performance of prediction models: a framework for some traditional and novel measures. *Epidemiology (Cambridge, Mass)* 2010;21(1):128.
35. Steyerberg EW, Vickers AJ. Decision curve analysis: a discussion. *Medical Decision Making* 2008;28(1):146-49.
