# Supplementary material for: Development and performance evaluation of the Medicines Optimisation Assessment Tool (MOAT): a prognostic model to target hospital pharmacists’ input to prevent medication-related problems
Source: BMJ Qual Saf. 2019 Mar 7;28(8):645–56. doi: 10.1136/bmjqs-2018-008335 (PMC6716361; doi:10.1136/bmjqs-2018-008335)
Supplement: Supplementary data [file bmjqs-2018-008335supp002.pdf]

S2 – Supplementary information on results

EXCLUDED ADMISSIONS

Further information on patient exclusions is given in Table S2.1.

Table S2.1 – Explanation for patient exclusions

<table>
<tr><th>Reason for exclusion</th><th>Explanation</th></tr>
<tr><td>No review by pharmacist (n=54)</td><td rowspan="2">Fifty two patients at Hospital A and 2 patients at Hospital B were excluded because they were not reviewed by a pharmacist during their admission. The number was higher at Hospital A due to the different method of patient identification used. The electronic bed record was used to identify new admissions at Hospital A, meaning that some patients were included in the study, but discharged before pharmacy staff visited the ward (later the same day). At Hospital B, eligible patients were identified by pharmacy staff once they visited wards.

One patient was excluded at Hospital A because they were admitted outside working hours. This occurred due to use of the electronic bed record system, which showed the patient as a current admission, but they were later identified as having been discharged prior to the start of the working day.</td></tr>
<tr><td>Admission outside working hours (n=1)</td></tr>
<tr><td>No medicines prescribed (n=12)</td><td>This included patients who were prescribed no medication (once only, when required or on a regular on-going basis) at any point during their hospital admission.</td></tr>
<tr><td>Not a medical patient (n=31)</td><td>This included patients admitted under the care of other specialities (e.g. surgery).</td></tr>
<tr><td>Admitted prior to start of study (n=11)</td><td>This included patients who were new to a medical ward during the study period rather than new to the hospital (e.g. initially admitted to a surgical ward).</td></tr>
<tr><td>Prescribing records unavailable (n=18)</td><td rowspan="2">Hospital A had electronic medical and prescribing records; Hospital B had paper based systems. As a result, some prescribing records/medical notes were unavailable for patients at Hospital B, which appeared to be due to misfiling.</td></tr>
<tr><td>Study admission not in medical notes / notes unavailable (n=17)</td></tr>
<tr><td>Elective admission (n=5)</td><td>This included patients admitted for investigation only.</td></tr>
</table>

MEDICATION RELATED PROBLEM (MRP) IDENTIFICATION ASSESSMENT EXERCISE

As stated in the main paper, the overall percentage agreement (proportion of MRPs identified) by 59 pharmacists from the study sites was 84.5%, with a Randolph's kappa coefficient of 0.50 suggesting 'moderate agreement'. It is possible that some of the observed variability in MRP identification was related to limitations in the assessment method, such as the choice of medication chart used for the assessment, but additional factors linked to the knowledge, experience and skills of study pharmacists also appeared to have an impact. For example, awareness of interactions, differences in professional judgement, and perceived likelihood of the error reaching the patient. This potential variability needs to be recognised as a limitation of the MRP data collection method, although prospective identification by pharmacy staff was chosen as it aligns with the proposed purpose of the Medicines Optimisation Assessment Tool (MOAT), which is to identify patients at risk of MRPs that can be identified during routine clinical practice.

TRUNCATION OF OUTLIERS

Table S2.2 gives the number (and percentage) of outliers for each predictor, with the range of values. The number of outliers per variable ranged from one data point (0.07%) for 'age', to 132 (8.8%) for 'number of hospital admissions in previous six months'.

Table S2.2 – Number and ranges for outliers

Predictor	Outlier(s)* (admissions = 1,503) n (% of admissions†)		Value / range of outlier(s)	
	Below lower truncation point	Above upper truncation point	Below lower truncation point	Above upper truncation point
Age (years)	1 (0.07)	0	17	N/A
Socioeconomic status, ranked using English Indices of Deprivation 2015 – Index of Multiple Deprivation[1]	0	0	N/A	N/A
Body mass index (kg/m^2)	0	30 (2.6)	N/A	40.8-65.5
Number of hospital admissions in previous 6 months	0	132 (8.8)	N/A	3-10
Number of comorbidities	0	16 (1.1)	N/A	10-13
Number of medicines prescribed	0	26 (1.7)	N/A	18-27
Renal function - estimated glomerular filtration rate‡ ($ml/min/1.73m^2$)	0	38 (2.5)	N/A	162-309
Serum albumin (g/L)	34 (2.3)	5 (0.3)	7-19	48-55
Serum potassium (mmol/L)	9 (0.6)	18 (1.2)	2.3-2.8	6-7.8

Continued from previous page…

Predictor	Outlier(s)* (admissions = 1,503) n (% of admissions†)		Value / range of outlier(s)	
	Below lower truncation point	Above upper truncation point	Below lower truncation point	Above upper truncation point
Serum sodium (mmol/L)	68 (4.5)	20 (1.3)	111-127	148-170
White cell count (10^9/L)	0	64 (4.3)	N/A	20.8-93.0
Platelet count (10^9/L)	3 (0.2)	51 (3.4)	5-7	492-977

N/A = not applicable

* Limit set at one and a half times the interquartile range above or below the third or first quartile respectively

† Calculated as the number of outliers / number of available values for each variable (i.e. excludes admissions with missing data)

‡ Glomerular filtration rate estimated using modified Modification of Diet in Renal Disease (MDRD) equation[2]

Plausibility of the outlying values was assessed by reviewing the distribution of each predictor across the full range of observed values. This found that all outlying results were clinically plausible, and consistent with the overall data distributions. No obvious data entry errors were identified therefore no data were set to missing.

The results of univariable logistic regressions using non-truncated and truncated data suggested that seven of the 12 predictors had outlying data points that were not representative of the remaining sample population: body mass index, number of previous hospital admissions, renal function, serum albumin, serum potassium, white cell count and platelet count. For example, truncation of 64 outlying results for ‘white cell count’, representing 4.3% of admissions, caused an increase in the regression coefficient of 125%; inclusion of these outlying data points in the analysis therefore had the potential to significantly alter the predictor-outcome relationship.

MISSING DATA

The unavailability of prescribing and/or medical records for 35 patient admissions (Figure 1) appeared to be due to misfiling of records. Data were therefore likely to be ‘missing completely at random’,[3] meaning participants were likely to be a truly random subset of the study sample.[4] Exclusion was therefore unlikely to bias the study results.

Of the 1,503 admissions included in the regression analyses, 387 (25.7%) had one or more missing predictor data point, accounting for 1.6% of the total candidate predictor data (see Table 1). A comparison of admissions with and without missing data suggested that data were ‘missing at random’ (MAR), as the admissions with missing data were clearly not a

random subset of the study sample.[3] This finding supports the use of multiple imputation to handle missing study data. The sensitivity analyses performed following multiple imputation provided no evidence against the MAR assumption, this is, there were plausible explanations for observed differences between multivariable regression estimates for the complete-case and imputed datasets.

DECISION CURVE ANALYSIS

The decision curve for the MOAT is shown in Figure 2. As anticipated, the 'treat none' and 'treat all' lines cross at the prevalence of the outcome event (40.6%). The MOAT is comparable to the strategy of 'treat all' at low threshold probabilities, and comparable to 'treat none' at high probabilities. This is because the probability of an outcome event predicted by the MOAT ranges from 9% to a maximum of 86%; using the MOAT below or above this range therefore gives the same result as 'treat all' or 'treat none'. Between approximately 70% and 85% the net benefit is approximately equal to the strategy of 'treat none', this is because of the relative increase in false positive compared to true positive results. Between approximately 15% and 70% the MOAT is better than both the 'treat none' and 'treat all' strategies, suggesting it is of value for threshold probabilities within this range.[5] The use of decision thresholds below 15% or above 70% would therefore offer no clinical benefit. Given that the decision thresholds (i.e. the minimum predicted risk probabilities to justify pharmacists' input) selected for the MOAT are 25% and 35%, both are within the range of threshold probabilities where the MOAT is considered to be clinically useful.

ASSESSMENT OF CLINICAL CREDIBILITY

Consensus views on the MOAT

The median score for each consensus statement, interquartile range (IQR), and range of scores are shown in Table S2.3. This shows that the median response and IQR were within the 'agreement category' for all four statements (i.e. within the range of 7-9).

Table S2.3 – Consensus scores of practising pharmacy staff on the clinical credibility of the MOAT (using nine-point Likert scale)

Consensus statement	Scores*		
	Median	IQR	Range
The choice of risk factors is appropriate	8	8 - 8	7 - 9
The presentation of the MOAT is reasonable	8	8 - 8	7 - 9
The MOAT is simple to interpret	8	8 - 9	8 - 9
The time taken to use the MOAT is reasonable	8	7 - 8	6 - 8

* A score of one indicates total disagreement, and nine indicates total agreement. A nine-point scale was used to permit the responses to be categorised; a score of one to three represents

disagreement, scores of four to six represent an equivocal response, and scores of seven to nine represent agreement.

IQR = interquartile range, MOAT = Medicines Optimisation Assessment Tool

Workload implications

Approximately 78% of patients in the developmental dataset 'screened positive' (i.e. were above the low/medium risk decision threshold), meaning that the MOAT permits identification of the 22% patients least likely to experience a moderate or severe preventable MRP.

The median time required to apply the MOAT was 2 minutes 18 seconds (range 1 minute 28 seconds to 5 minutes 4 seconds; IQR 1 minute 41 seconds to 3 minutes 12 seconds).

Clinical implication of false negative predictions

There was evidence to suggest that the 61 patients who experienced moderate or severe preventable MRPs despite being categorised as low-risk (false negatives) experienced fewer outcome events (p=0.0021) that were of lower severity (p=0.046), compared to 545 patients categorised as medium or high-risk (true positives).

REFERENCES

1. GOV.UK. English indices of deprivation 2015, 2015.
2. Michels WM, Grootendorst DC, Verduijn M, et al. Performance of the Cockcroft-Gault, MDRD, and new CKD-EPI formulas in relation to GFR, age, and body size. *Clinical Journal of the American Society of Nephrology* 2010;5(6):1003-09.
3. Sterne JA, White IR, Carlin JB, et al. Multiple imputation for missing data in epidemiological and clinical research: potential and pitfalls. *Bmj* 2009;338:b2393.
4. Moons KG, de Groot JA, Bouwmeester W, et al. Critical appraisal and data extraction for systematic reviews of prediction modelling studies: the CHARMS checklist. *PLoS Med* 2014;11(10):e1001744.
5. Vickers AJ, Elkin EB. Decision curve analysis: a novel method for evaluating prediction models. *Medical Decision Making* 2006;26(6):565-74.
