# Supplementary figures and images for: Development and performance evaluation of the Medicines Optimisation Assessment Tool (MOAT): a prognostic model to target hospital pharmacists’ input to prevent medication-related problems
Source: BMJ Qual Saf. 2019 Mar 7;28(8):645–56. doi: 10.1136/bmjqs-2018-008335 (PMC6716361; doi:10.1136/bmjqs-2018-008335)

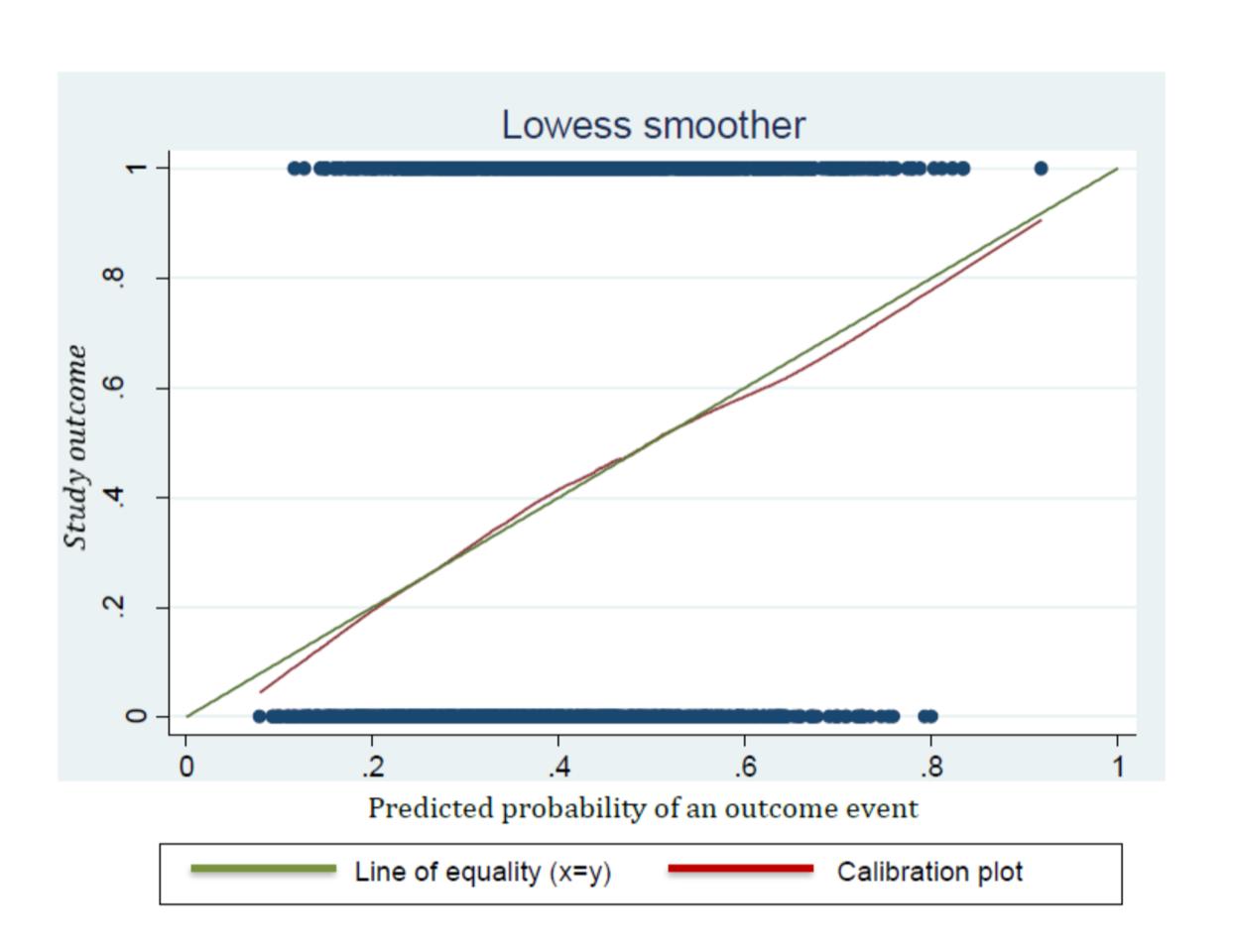

Supplement: Supplementary data [file bmjqs-2018-008335supp003.jpeg]
