# Supplementary material for: Development and performance evaluation of the Medicines Optimisation Assessment Tool (MOAT): a prognostic model to target hospital pharmacists’ input to prevent medication-related problems
Source: BMJ Qual Saf. 2019 Mar 7;28(8):645–56. doi: 10.1136/bmjqs-2018-008335 (PMC6716361; doi:10.1136/bmjqs-2018-008335)
Supplement: Supplementary data [file bmjqs-2018-008335supp004.pdf]

S4 – Full regression equation for MOAT

The predicted risk for an individual patient can be calculated using the standard logistic regression equation:

$$\text{Log-odds of the outcome event} \quad = \beta_0 + \beta_1 x_1 + \beta_2 x_2 + \cdots + \beta_p x_p$$

where β_0 is the constant. The quantity on the right-hand side of the equal sign is the linear predictor of the log-odds of the outcome given the particular value of the p exposure variables x_1 to x_p (where x takes the numerical value of continuous variables, and is coded zero or one for categorical variables). The β's are the regression coefficients associated with the p exposure variables.

The logistic regression model is a non-linear transformation of the linear regression and it ensures that predicted probabilities lie between 0 and 1. The predicted probability can be calculated by exponentiating the log-odds (to give the odds of the outcome event), followed by use of the formula:

Predicted probability = $\frac{odds}{(1 + odds)}$

Alternatively, predicted probability = $\frac{1}{(1 + \exp(-(log\ odds))}$

The predicted probability of experiencing at least one moderate or severe preventable medication related problem can therefore be calculated using the following formula:

Probability = 1/(1 + exp(-(-1.674 + 0.125 x number of comorbidities - 0.0308 x estimated glomerular filtration rate*/10 + 0.0234 x white cell count* + 0.0347 x number of medicines + 0.272 x previous allergy + 0.354 x nervous system and mental disorders - 0.234 x respiratory system - 0.533 x gastrointestinal system + 0.331 x aminoglycosides and glycopeptides + 0.311 x other antimicrobials + 0.385 x epilepsy medicines)))

* Truncated value should be used if actual result above the following truncation point:

- estimated glomerular filtration rate = 161 ml/min/1.73m^2;
- white cell count = 20.7 10^9/L.

Please note, categorical variables such as 'previous allergy' should be coded as 'one' if present and 'zero' if absent (see Table 2).
